# Occupational noise exposure and hearing: a systematic review

**DOI:** 10.1007/s00420-015-1083-5

**Published:** 2015-08-07

**Authors:** Arve Lie, Marit Skogstad, Håkon A. Johannessen, Tore Tynes, Ingrid Sivesind Mehlum, Karl-Christian Nordby, Bo Engdahl, Kristian Tambs

**Affiliations:** National Institute of Occupational Health, P.O. Box 8149 Dep., 0033 Oslo, Norway; Norwegian Institute of Public Health, Oslo, Norway

**Keywords:** NIHL, ISO, Population studies, Vibration, Cardiovascular risk factors, Chemicals, Leisure-time noise, Mechanisms

## Abstract

**Purpose:**

To give a systematic review of the development of noise-induced hearing loss (NIHL) in working life.

**Methods:**

A literature search in MEDLINE, Embase, Web of Science, Scopus, and Health and Safety Abstracts, with appropriate keywords on noise in the workplace and health, revealed 22,413 articles which were screened by six researchers. A total of 698 articles were reviewed in full text and scored with a checklist, and 187 articles were found to be relevant and of sufficient quality for further analysis.

**Results:**

Occupational noise exposure causes between 7 and 21 % of the hearing loss among workers, lowest in the industrialized countries, where the incidence is going down, and highest in the developing countries. It is difficult to distinguish between NIHL and age-related hearing loss at an individual level. Most of the hearing loss is age related. Men lose hearing more than women do. Heredity also plays a part. Socioeconomic position, ethnicity and other factors, such as smoking, high blood pressure, diabetes, vibration and chemical substances, may also affect hearing. The use of firearms may be harmful to hearing, whereas most other sources of leisure-time noise seem to be less important. Impulse noise seems to be more deleterious to hearing than continuous noise. Occupational groups at high risk of NIHL are the military, construction workers, agriculture and others with high noise exposure.

**Conclusion:**

The prevalence of NIHL is declining in most industrialized countries, probably due to preventive measures. Hearing loss is mainly related to increasing age.

**Electronic supplementary material:**

The online version of this article (doi:10.1007/s00420-015-1083-5) contains supplementary material, which is available to authorized users.

## Introduction

Hearing loss due to noise exposure in the workplace is a significant health problem with economic consequences. Noise-induced hearing loss (NIHL) is the occupational disease most frequently reported to the Norwegian Labour Inspection Authority and the Petroleum Safety Authority. Every year the two authorities receive close to 2000 and 600 new reports of NIHL, respectively, accounting for 60 % of all reported work-related diseases (Samant et al. 2008) in a working population of 2.7 million.

NIHL is also regarded as a serious problem and one of the most recorded occupational disorders in Europe and in the rest of the world and amounts to between 7 and 21 % of the hearing loss (Nelson et al. 2005; Dobie 2008). While the incidence of NIHL seems to decrease in other European countries (EASHW 2005), the figures have been stable in the Norwegian mainland sector for the last 20 years and increasing in the Norwegian offshore sector, in spite of comprehensive preventive measures.

On this background, the Norwegian Ministry of Labour and Social Affairs requested the National Institute of Occupational Health to conduct a systematic literature review of occupational noise exposure and health. This paper reviews the literature on occupational noise exposure and hearing.

## Methods

We searched the following databases for peer-reviewed studies on occupational noise and hearing loss, and other health outcomes: Ovid MEDLINE (1946–), Ovid Embase (1974–), Web of Science (1950–), Scopus (1995–), and ProQuest Health and Safety Sciences Abstracts (1981–). A search strategy was developed for each database. In the databases that are indexed by a hierarchical controlled vocabulary (MEDLINE and Embase), we used a combination of free text terms and controlled vocabulary (MeSH and EMTREE). The search strategy was developed with low specificity to the advantage of high sensitivity, i.e., high probability of hits on potentially relevant studies. The search was completed in May 2013. For more information, see the supplementary file.

### Inclusion and exclusion criteria

Inclusion criteria were exposure to occupational noise alone or in combination with other factors, hearing loss and other health outcomes and the statistical association between occupational noise and hearing loss/other health outcomes. Papers in other languages than in English and animal studies were excluded.

All titles and abstracts from the literature search were assessed against the inclusion criteria for possible relevance. References that we judged to be potentially relevant were read in full text and evaluated. Relevant original studies (698 papers) were quality-assessed by at least one researcher using a comprehensive checklist for observational studies developed by the National Institute of Occupational Health based on the checklist of Ariëns et al. (Ariens et al. 2000) and Hoogendoorn et al. (1999).

The checklist consisted of two parts:

1. Internal validity related to the study population, exposure and outcome measures, data analysis, data presentation and control for confounding.
2. External validity related to the representativeness of the study populations.

**Fig. 1:**
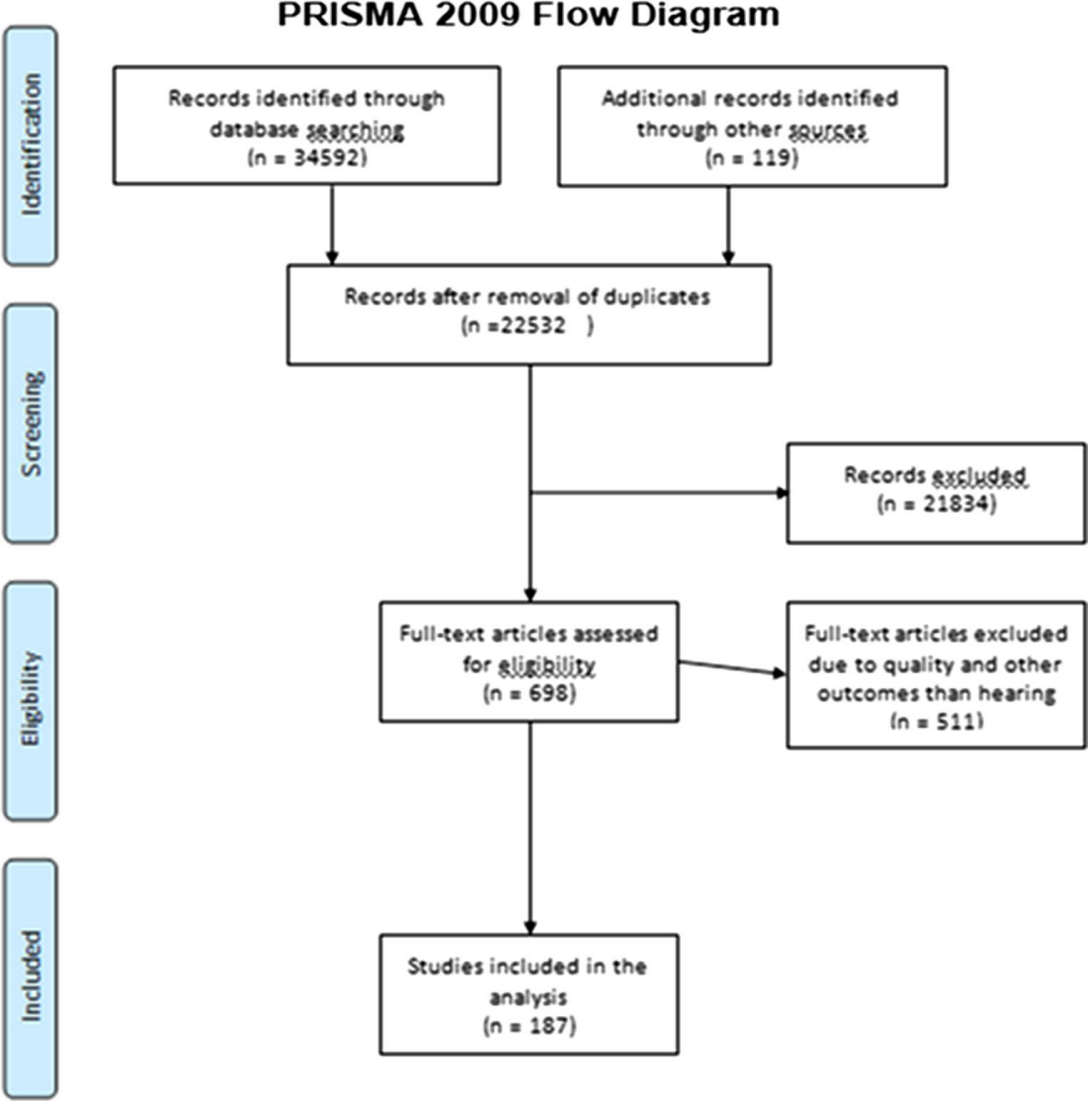
Prisma flow diagram for the study

As to the present review, only articles regarding noise and hearing but not other health outcomes were included, both cross-sectional and longitudinal studies. A total of 187 papers met the inclusion criteria. These were cross-sectional studies (*N* = 106), longitudinal studies (*N* = 52), reviews (*N* = 22) and others (*N* = 7). Figure 1 is the prisma flow diagram of the study.

## Results

### Noise-induced hearing loss: different definitions

There are several audiological definitions of noise-induced hearing loss. Some focus on hearing loss in the frequency range 0.5–2 kHz and some on the range from 0.5 to 4 kHz, while others again put emphasis on high-frequency hearing loss from 3 to 6 kHz. Some definitions calculate the hearing loss as the average of both ears, others for the better ear and still others for the worse ear. The criteria for occupational noise-induced hearing loss also vary from country to country (Rabinowitz 2012). Thus, the divergent outcome measures used in previous research make it difficult to compare the results.

### Normal hearing

Initially, studies describing normal hearing should be mentioned. It is not possible to assess causes of a hearing loss without a reference for comparison. ISO 1999 is an international standard based on US studies from the 1960s and the 1970s (ISO 1990a, b) describing what is a normal hearing threshold for both sexes at various ages. The standard indicates that the hearing threshold increases with age in the frequency range of 3–8 kHz and that women lose less hearing than men. Annex A of the standard tabulates the expected hearing loss in a highly screened population, where people with noise exposure and ear disease have been removed. In Annex B, both of these groups are included; accordingly, the expected hearing loss is greater in Annex B than in A.

**Fig. 2:**
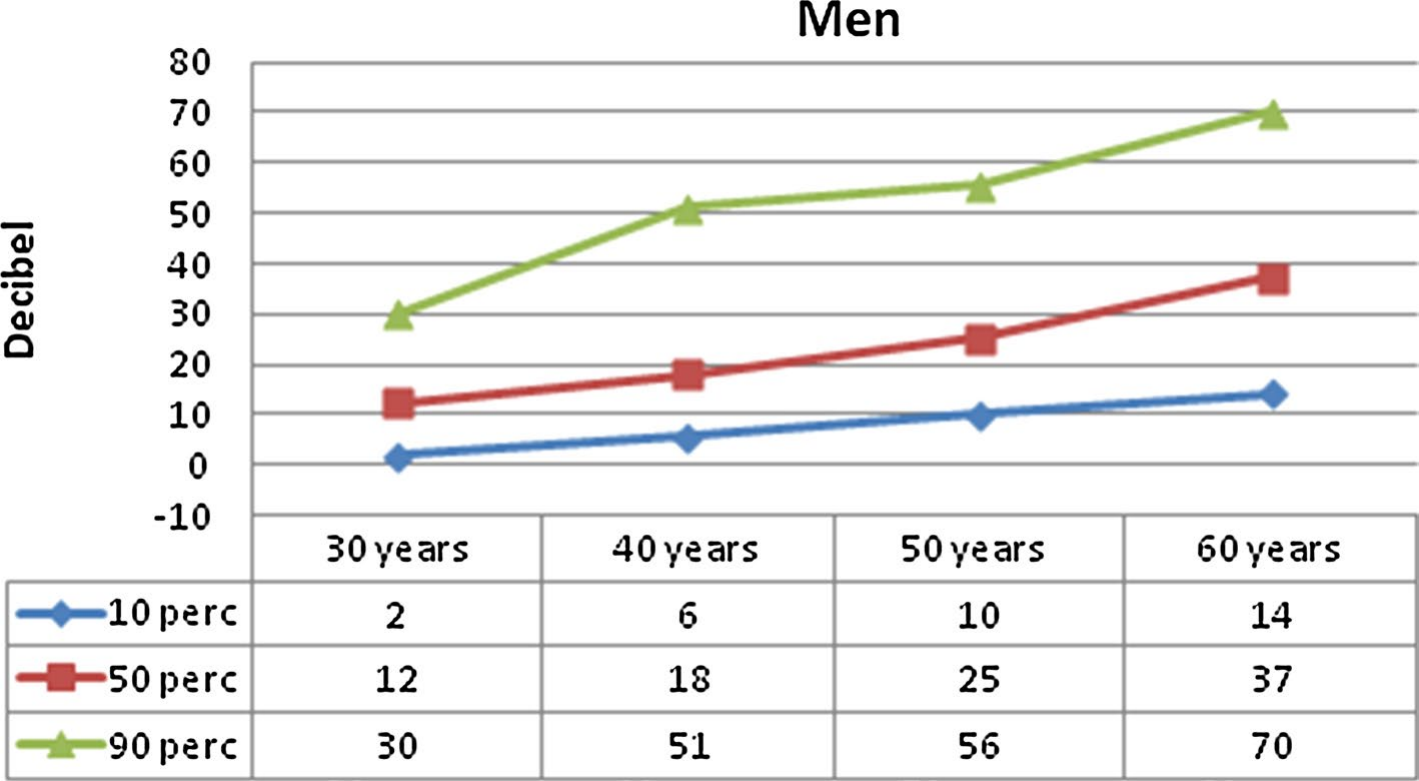
Expected hearing threshold in men, 10, 50 and 90 percentile, 3–6 kHz, better ear by age. Based on ISO 1999 (1990), Annex B

**Fig. 3:**
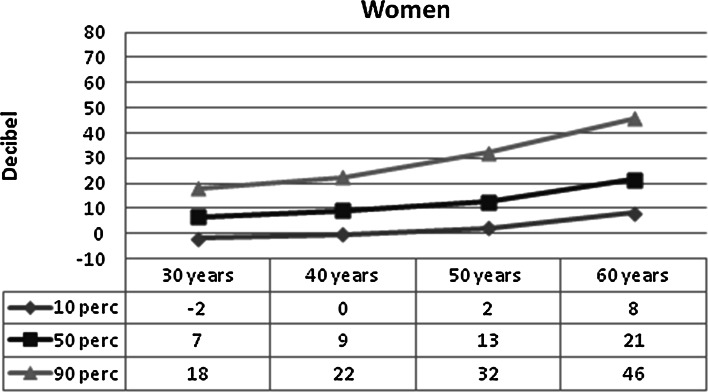
Expected hearing threshold in women, 10, 50 and 90 percentile, 3–6 kHz, better ear by age. Based on ISO 1999 (1990), Annex B

Figures 2 and 3 show the mean hearing threshold in the “noise-sensitive area” of 3–6 kHz, in men and women, respectively, in relation to age. The figures are based on ISO 1999, Annex B. The hearing threshold increases markedly with age, but the individual differences are large. The hearing thresholds are highest in men. The median hearing threshold for a 60-year-old is 37 dB for men and 21 dB for women, but the central 80 % distribution spans some 40 dB in women and 55 dB in men.

**Fig. 4:**
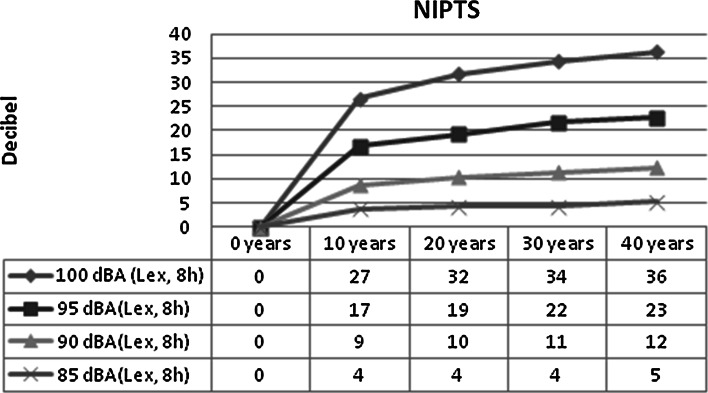
Noise-induced permanent threshold shift (NIPTS) by noise exposure level and years of exposure. Men and women. Based on ISO 1999: 1990, Annex E

There are also reference values of the hearing loss at different frequencies for different levels of noise exposure (ISO 1999, Annex E) (ISO 1990a, b). Most of the hearing loss occurs during the first 10 years of noise exposure. For example, the median expected noise-induced permanent threshold shift (NIPTS) of 3–6 kHz at a 85-dB noise exposure level normalized to a nominal 8-h working day (daily noise exposure level, *L*_ex, 8h_) for 10 years is 4 dB (2–5 dB for the 10–90 percentile) and after 40 years 5 dB (3–7 dB for 10–90 percentile) (Fig. 4). This means that the expected noise-induced hearing loss at a daily noise exposure level of 85 dB will be small compared with the age-related hearing loss. The NIPTS after 40 years of exposure at *L*_ex, 8h_ = 100 dB of 3–6 kHz will be 36 dB. (The actual NIPTS, however, will be somewhat smaller since it should be adjusted according to the term (*N* − *H* × *N*/120), where *N* is NIPTS and *H* the expected age-related permanent threshold shift (ISO 1990a, b). In a 60-year-old man with a noise exposure at *L*_ex, 8h_ = 100 dB, the actual NIPTS of 3–6 kHz will be reduced from 36 dB down to 25 dB.)

Hearing loss is often familiar. Twin studies (Karlsson et al. 1997; Christensen et al. 2001; Viljanen et al. 2007; Wingfield et al. 2007) and studies of siblings and parent–child (Gates et al. 1999; Raynor et al. 2009; Demeester et al. 2010) have shown that genetic predisposition is very important for hearing. Most studies show that between half and two-thirds of the variation in hearing acuity, adjusted for age, can be attributed to individual differences in genetic susceptibility. Many of the studies, however, are of small or moderate size, and the results differ substantially. A large sample study of siblings by Kvestad et al. shows a heritability (the proportion of variation in the population attributable to genes) of approximately 0.4 (Kvestad et al. 2012).

In recent years, there have also been a number of molecular genetic studies of the relationship between genetics and hearing impairment (Carlsson et al. 2005; Chang et al. 2009, 2011; Konings et al. 2009; Lin et al. 2009; Pawelczyk et al. 2009; Liu et al. 2010; Shen et al. 2012; Li et al. 2013). Genes involved in the management of oxidative stress, endolymphatic potassium transport and “heat shock” proteins have been most studied. As of today, however, there is no genetic test that can distinguish between those who are susceptible or resistant to noise-induced hearing loss.

### Occupations and hearing

We identified 96 published studies describing hearing loss in various professions: 65 cross-sectional, 27 with a longitudinal design and 4 review articles.

Generally the quality of the hearing data is good, while the noise exposure data are often blunt or incomplete. In the following, we have highlighted some research on various specific professions to identify the relationship between occupation and hearing.

#### Industrial workers

In a Swedish cross-sectional study, Ivarsson et al. compared hearing in employees in the automotive industry, shipyards and quarries (*N* = 1796) (*L*_ex, 8h_ > 95 dB) for the period 1983–1990 with hearing in office workers (*L*_ex, 8h_ < 80 dB) and found that among blue collar workers over the age of 50, only 8–28 % had a normal hearing, compared to 70 % among office workers (Ivarsson et al. 1992). The results were compared with another Swedish study from 1970 to 1971. Exposed workers more than 50 years had about the same level of hearing acuity in the 1980s as in the 1970s, while exposed workers aged 20–30 had a significantly better hearing in the 1980s compared to the 1970s and about as well as nonnoise-exposed workers.

In a longitudinal study for the period 1983–1989, Lee-Feldstein compared the hearing loss in the range 2–4 kHz of 11,435 US automobile workers in five plants over a 5-year period with 331 nonexposed controls (Lee-Feldstein 1993). The aim of this study was to evaluate the efficacy of preventive measures. The noise exposure was from *L*_ex, 8h_ = 85 to 114 dB. In four of the five factories, the difference in hearing between exposed and controls was not significant.

In a retrospective study of male metal workers at a Swedish car factory, the hearing loss for 1964, 1972, 1980, 1987 and 1989 was compared to ISO 1999, Annex A (Bruehl et al. 1994). The study showed that during the period 1964–1989, the hearing loss decreased from about 20 to 5 dB in the age group of 20–29 years and from 30 to 10 dB in the age group 50–59 years, all values compared to ISO 1999 A. The authors attributed this improvement to better access to and use of hearing protection and lower noise exposure during the period. If the results had been compared to recent Swedish reference values (Johansson 2002) instead of the ISO 1999 Annex A, hearing among the youngest in 1989 would have been found close to normal.

Hearing loss was also found in a Russian study of the personnel in two workshops, revealing that impulse noise caused a greater hearing loss than one would expect from the estimated A-weighted noise dose (Suvorov et al. 2001). The authors suggested that an extra safety margin of 5 dB should be subtracted from the threshold value for what is considered harmful impulse noise.

Martin et al. (1975) examined hearing in 228 Canadian smelter workers in three different departments and compared it to nonexposed employees from the same factory. The daily noise exposure level was mostly below 90 dB, which was then the Canadian threshold limit value (TLV), and therefore hearing protection was not found to be necessary. The prevalence of a hearing loss, defined as the average >25 dB for the 0.5–2-kHz area, ranged from 14 to 32 % in exposed >50 years, compared to 4 % of the controls. The hearing loss was greatest in areas with impulse noise and lowest in areas with a continuous noise level, *L*_Aeq_, of 85–90 dB.

Keatinge and Laner examined 63 factory workers younger than 40 years with a noise exposure, from 115 to 128 dB (sound pressure level, SPL), without the use of hearing protection, and with short service time, and compared their hearing with hearing in a control group. They found that most of the hearing loss occurred during the first 3 years of employment (Keatinge and Laner 1958).

Noise-exposed industrial workers (*N* = 47,388), 85 % men, mostly between 20 and 55 years, and with a daily noise exposure level between 86 and >105 dB, were examined in Austria. The strongest estimated effect of noise, adjusted for age, gender and other covariates, was a standardized regression coefficient of 0.08 for 4 kHz (Bauer et al. 1991). This corresponds to a 5-dB difference between nonexposed and heavily exposed subjects.

In a cross-sectional study, Somma et al. (2008) examined hearing of 184 male cement workers, with a daily noise exposure level >85 dB and compared it with 98 nonexposed controls. The study revealed a hearing loss in the 3–6-kHz range of 5 dB among the youngest, age 21–30, and 20 dB among the oldest, age 51–60, compared to the controls.

In a follow-up study of 449 male steel workers with exposures ranging from *L*_Aex, 8h_ < 90 to *L*_Aex, 8h_ > 100 dB, no changes in hearing in the 0.5–6-kHz range during a 6–8-year period could be attributed to noise exposure (Howell 1978).

In a longitudinal study of 113 Egyptian male cotton workers, Moselhi et al. (1979) reported that a daily noise exposure level <85 dB gave only a slight hearing loss compared to the control group (*N* = 64), whereas exposure >85 dB revealed a hearing loss of >25 dB in the frequency range 0.5–2 kHz in 9.6 % of the employees.

Swedish pulp workers (*N* = 319), aged 26–40 years at the beginning of the study, with varying daily noise exposure levels from 80 to 100 dB, were followed with repeated audiometric examinations from 1959 and 20 years ahead. The hearing loss was most pronounced at 4 kHz, approximately 15 dB. There was only a small difference in hearing between high- and low-exposed subjects. The authors suggested that the small difference might reflect chemical exposure among the workers with low noise exposure. The importance of age as a possible cause of the hearing loss was not discussed in this study (Bergstrom and Nystrom 1986).

Egyptian metal workers (*N* = 88) were examined in 1980 and then again 8 years later. They were exposed to a mean background noise, *L*_Aeq_, of 90–94 dB and impulse noise, *L*_A, peak_ of 112–139 dB with 20–50 “beats per minute” (bpm). They did not use any hearing protection, and the hearing loss was significant in 1980. Upon follow-up, the age-adjusted hearing loss had increased somewhat, particularly in the 0.5–2-kHz range. Most of the hearing loss in the higher frequencies (3–8 kHz) occurred in the first 10–15 years after employment (Kamal et al. 1989).

Rabinowitz et al. followed the hearing acuity in 6217 aluminum workers with daily noise exposure in four categories from <82 to >88 dB during the period from 1990 to 1996. The hearing loss in the period was lowest for the highest exposed group and normal for the lowest exposed group, compared with US reference values. The use of hearing protection among the highest exposed may partly explain the findings (Rabinowitz et al. 2007).

Hearing in 78 male aluminum workers with daily noise monitoring inside the ear protectors was followed for 4 years and compared with a control group (*N* = 234) matched for age, initial hearing thresholds and noise exposure. The intervention group did not develop any hearing loss, while the control group who received no intervention, had a small hearing loss. The study shows that a close follow-up with the use of hearing protection may have a beneficial effect (Rabinowitz et al. 2011).

It appears that in Western countries, the noise exposure levels in the industry have been reduced over the last few decades and this has led to a reduced hearing loss and improved hearing in noise-exposed groups in recent years (Rubak et al. 2006). The hearing loss appears to be greatest during the first years of noise exposure. There are conflicting results as regards the effect of hearing protection, but a recent US study indicates that a close follow-up of the use of hearing protection has a clear advantageous effect on hearing (Rabinowitz et al. 2011).

#### Shipyard workers

In an Indian study, 276 shipyard employees with daily noise exposure >90 dB were compared with 276 age- and sex-matched controls from the office staff without any occupational noise exposure. Among the exposed workers, 6 % were assessed to have a NIHL, as compared to none of the controls. The criterion for NIHL was a notched hearing loss (Bhumika et al. 2013).

Nilsson et al. examined hearing of 1492 employees at a shipyard with an exposure to noise from *L*_Aeq_ = 88–94 dB and with a lot of impulse noise, typically 2500 impulses per day with peak noise levels from 105 to 135 dB (A). Despite the fact that almost 90 % used hearing protection, about 60 % had a reduced hearing acuity. After individual age correction to the ISO 1999, there were still about 40 % with reduced hearing acuity, most of them with exposure >5 years. The authors conclude that the noise in shipyards with impulse noise is particularly damaging to hearing (Nilsson et al. 1977).

Historically, there have been noise exposure levels in the shipyards high enough to cause NIHL.

#### Construction industry workers

Seixas et al. studied the hearing acuity of 393 apprentices in the building trades and 62 controls (students) from 2000 to 2010. At the last follow-up, the group was reduced to 258 in the building trades and 58 controls. The daily noise exposure level was 87 dB for the noise exposed and 70 dB for the students. An exposure increase of 10 dB gave a hearing loss of 2–3 dB after 10 years at 3–6 kHz. Most of the hearing loss had already occurred before the initial examination. Self-reported data on noise outside work had no impact on hearing. The author suggests that the limit of 90 dB does not assure good enough protection to prevent NIHL (Seixas et al. 2004, 2005, 2012).

Dutch construction workers (*N* = 29,644) with a daily noise exposure of 87–96 dB were examined longitudinally with respect to hearing and compared with an internal unexposed group. The hearing loss at 4 kHz ranged from 0 dB for the age group <25 years to about 7 dB for the 55–64 year group, compared with the internal control group, which corresponds very well to ISO 1999, Annex B (unscreened) (Leensen et al. 2011).

Engdahl and Tambs found that male construction workers may be one of the groups with the most pronounced NIHL with an average hearing loss of 9 dB in the 3–6-kHz frequency range and 6 dB for the average of 0.5, 1, 2 and 4 Hz, compared to nonnoise-exposed male teachers (Engdahl and Tambs 2010).

Overall, the literature indicates that NIHL is a frequent diagnosis in the construction industries, but we have found surprisingly few high-quality studies published from the industry. This is unfortunate, considering the potentially high noise exposure levels.

#### Offshore workers (oil and gas production at sea)

Morken et al. examined the incidence of NIHL among offshore workers on the Norwegian continental shelf, reported to the Petroleum Safety Authority (PSA) from 1992 to 2003. The study revealed a significant increase, from 1/1000 employed in 1992 to 9/1000 in 2003 (Morken et al. 2005). The majority of the cases were reported among mechanics, surface treatment workers, electricians, process technicians and roughnecks, most of them aged 50–59 years.

In 2002, Zacchariasen and Knusden stated that there is a problem with high noise exposure in the Norwegian offshore industry (Zachariassen and Knudsen 2002). Nistov et al. (2012) in a later study reported that there is a high noise exposure level, a risk of NIHL and a need for preventive measures in this industry.

Ross et al. (2010) however, found in a recent study that offshore workers except for divers had a normal hearing compared with a nonexposed population and so did Johnson and Gann (1991) in a former longitudinal study.

There is a great deal of concern over the noise exposure level and the perceived risk of NIHL in the offshore sector. The number of studies is limited, but the evidence suggests that offshore workers as a group have a relatively normal hearing. More and larger longitudinal studies are needed.

#### Professional divers

Previous cross-sectional studies of divers have not found any extra hearing loss compared to the general population, despite noise exposure, long diving experience and experienced barotrauma (Brady et al. 1976). More recent prospective studies of professional divers have shown that diving can cause impaired hearing, however (Molvaer and Lehmann 1985; Molvaer and Albrektsen 1990; Skogstad et al. 2000). Even among divers who do not report that they have been exposed to noise, excessive hearing loss has been found (Edmonds 1985). In most of these studies, exposure to noise has not been measured, but self-report and the number of dives are used as a proxy measure of exposure.

Divers can be exposed to both airborne and waterborne noise during their work, and high noise levels in the working environment of the diver can affect hearing (Summitt and Reimers 1971). It is very difficult to measure the noise submerged (Nedwell and Parvin 1994), and hearing under water is more characterized by bone conduction than air conduction (Hollien 1993). Since the impedance between water and air is different, the sound intensity will be lower in water at a given sound level. Noise from the air supply to the helmet, in addition to the noise from the pressure chamber and hydraulic tools, will add to the total noise exposure (Molvaer and Gjestland 1981; Curley and Knafelc 1987). Use of tools and explosions can generate impulse noise, even in water.

Solvents and gases, such as carbon monoxide, and heavy metals, such as lead, arsenic and mercury, might also affect hearing in divers (Phaneuf and Hetu 1990). Moreover, decompression sickness and barotrauma may be detrimental to hearing (Edmonds et al. 1992). Decompression sickness may occur by the formation of gas bubbles in the small blood vessels and the fluid in the inner ear (Shupak et al. 1991). Barotrauma of the inner ear may occur due to problems equalizing the pressure in the middle ear. This can happen during diving, when increased ambient pressure leads to a relatively low pressure in the middle ear (Edmonds et al. 1992). In addition, head injury and infections of the ear by Pseudomonas aeruginosa may affect hearing in divers (Edmonds et al. 1992; Ahlen et al. 1998). Hearing loss in the low-frequency ranges, 0.5–2 kHz, is described in two longitudinal studies among divers with and without noise exposure (Harashima and Iwasaki 1965; Haraguchi et al. 1999). In both cross-sectional and prospective studies, hearing loss in the high-frequency area has been associated with exposure in terms of number of dives (Coles 1976; Molvaer and Lehmann 1985; Zulkaflay et al. 1996; Skogstad et al. 2009; Ross et al. 2010). Some studies of divers’ hearing have revealed better hearing than the reference group even after 6 years of follow-up (Molvaer et al. 1983; Molvaer and Lehmann 1985; Skogstad et al. 2005). This may be due to selection mechanisms, as divers generally have better health and hearing than others due to screening procedures before hire.

Overall, there is evidence that diving can cause a moderate hearing loss in both the low- and the high-frequency ranges. This applies to both divers using air as the breathing gas and saturation divers. In the early years of the career, it appears that divers hear better than average. This may be due to health selection.

#### Fire fighters

In a cross-sectional study of firefighters in Phoenix and Fort Worth, USA, Clark and Bohl found that firefighters with an estimated equivalent noise exposure level of 80–90 dB (A) had a normal hearing, compared to ISO 1999. A follow-up study showed that the hearing loss in this group was somewhat less than expected from the ISO 1999 (Clark and Bohl 2005).

Similar findings were reported in a small study of 171 Korean firefighters with exposure to noise from emergency vehicles at *L*_ex, 8h_ = 99–108 dB (Kim et al. 2011) and in a study of 100 Iranian firemen who were compared with 100 nonexposed controls (Assadi et al. 2013).

Kales et al. (2001) compared hearing in 319 firefighters with ISO 1999, Annex A. Firefighters <40 years old had a nearly normal hearing, while the oldest (>50 years) had a hearing loss of 20–30 dB more than expected in the 3–6-kHz range.

Since younger firefighters seem to have a normal hearing, there is no basis for claiming that this group nowadays has a great risk of hearing loss caused by noise exposure in the workplace, but high-quality longitudinal studies are missing.

#### Military workers

A cross-sectional study of the incidence of hearing loss and tinnitus in 204 infantry officers exposed to impulse noise from various weapons with peak levels up to 185 dB (SPL), revealed a significant hearing loss compared with ISO 1999 (Christiansson and Wintzell 1993).

Segal et al. studied the development of hearing in 841 men aged 20–40 years with terminated exposure, but who had been exposed to noise trauma (shooting/explosions) and who had applied for financial compensation. Hearing in this group was compared with hearing in 150 men with continued exposure. Hearing in the noise trauma group stabilized after approximately 1 year, whereas the group with continued exposure had a continued hearing loss (Segal et al. 1988).

In three large studies of 87,000 to over 140,000 US military personnel, Helfer et al. (2005, 2010) and Helfer (2011) reported a higher than expected incidence of hearing loss among infantrymen, in those with active war experience, in men compared to women and in those over 40 years. Unfortunately, high-quality exposure data are missing.

In a prospective study of 804,535 soldiers returning from Iraq and Afghanistan in 2003–2009, the incidence of reported cases of hearing loss increased significantly during the period, from no cases in 2006 and earlier to about 5 cases per 100 in the first quarter of 2009 (Helfer et al. 2011). This was attributed to increased focus on hearing loss since 2006, showing that the extent of hearing loss may be significant for military missions.

Similar findings have been reported from a follow-up hearing study of 747 recruits with a service time of 11.7 months, who were compared with nonexposed controls (Muhr et al. 2006). Of the artillery recruits, 17 % had a “significant hearing loss” of >15 dB in at least one frequency, compared to 2.9 % of the controls. The authors conclude that recruits still lose hearing during service despite comprehensive preventive measures.

Hearing in pilots in the Finnish Air Force was examined by Kuronen et al. (2004) in a cross-sectional study. The equivalent noise exposure level in the cockpit was 90–100 dB (A), but the exposure periods were relatively short. Comparing with ISO 1999, the pilots heard clearly better than normal. Health selection criteria for recruitment to the pilot profession were the most probable cause.

Ribak et al. (1985) reported that hearing in 777 pilots/navigators in the Israeli Air Force was reduced with increasing age, but no significant impact of the type of aircraft or the number of hours flown could be demonstrated.

Hearing in 525 pilots aged 20–40 years in the French Air Force was examined in a cross-sectional study (Raynal et al. 2006). “Abnormal hearing” was detected in 19 % of the 20–30-year-old pilots and 38 % in those aged 30–40 years. Transport pilots had a slightly better hearing than the fighter and helicopter pilots. All groups had an audiometric notch at 6 kHz. Their hearing was not compared to any reference material, and noise exposure data were not provided.

In a prospective study, 512 pilots in the French Air Force aged 20–40 years, exposed to a noise level from 90 to 140 dB (A) (SPL), were followed over a 3-year period. Hearing was measured by pure tone audiometry and otoacoustic emissions (OAE) (Job et al. 2009). The author concluded that the use of OAE to some extent could predict who was at risk of developing reduced hearing.

Trost and Shaw (2007) compared the first and last audiograms of 267,568 persons in the US Navy during the period 1982–2004 and found that the risk of developing a hearing loss of 10 dB or more in the 2–4-kHz range was increased more for every year serving on a warship (relative risk, RR 1.062) compared to service ashore (RR 1.035). The daily exposure level was >84 dB.

Overall, it appears that the military work experience is a significant risk factor for hearing impairment.

#### Civil aviation workers

In a prospective study, Wagstaff and Årva compared hearing in Norwegian pilots and helicopter pilots over a 2–3-year period with air traffic controllers and reference material (ISO 1999). The hearing loss in the 3-, 4- and 6-kHz ranges was slightly greater than expected according to ISO, but not compared to the air traffic controllers. Helicopter pilots had a normal hearing despite a significantly higher noise exposure, *L*_Aeq_, of 90–95 dB, compared to 80–85 dB for fixed wing pilots (Wagstaff and Årva 2009).

Qiang et al. followed 3019 pilots aged 45–54 years flying smaller aircrafts over a 10-year period. The noise exposure in smaller aircrafts was assessed to be slightly higher than in ordinary civil aviation. The flight time was used as a proxy measure of cumulative noise exposure. When adjusted for age, there was a positive trend for the effect of flying hours, but it was not statistically significant (Qiang et al. 2008).

In a cross-sectional study, Kidera and Gaskill compared hearing in 1443 US airline pilots with a reference material. The pilots had a normal hearing (Kidera and Gaskill 1974).

Lindgren reported that hearing in Swedish pilots (*N* = 664) and cabin crew (*N* = 936) with a daily noise exposure level of 75–81 dB was normal, compared to a Swedish reference population (Lindgren et al. 2008, 2009).

In a cross-sectional study, Smedje et al. compared hearing in 327 aircraft mechanics with a Swedish reference population. The daily noise exposure was from 70 to 91 dB with A-weighted maximum levels at 119 dB (Smedje et al. 2011). A median hearing loss of 2–3 dB in the group of 35–39 years was found. Other age groups had normal or slightly better than normal hearing.

Overall, civil aviation personnel with the current hearing conservation measures are generally not exposed to a noise level that is detrimental to hearing.

#### Railway workers

In a cross-sectional study of 9778 male railway employees, a hearing loss of 2–7 dB was found in the frequency range above 2 kHz, compared to ISO 1999, Annex B (Kryter 1991). The use of firearms was reported to be associated with a greater hearing loss. In another cross-sectional study, Clark and Popelka (1989) found that hearing in 9427 trainmen did not differ from that of a reference population (ISO 1999). The daily noise exposure was on average 78 dB and ranged from 61 to 89 dB.

Virokanna et al. (1994) found that Finnish railway track maintenance workers with a daily noise exposure level of 94 dB (A) had a smaller hearing loss than expected, compared to the ISO 1999. This was attributed to the use of hearing protection.

Overall, there is no basis for claiming that this group runs a high risk of hearing loss caused by noise exposure in the workplace, but longitudinal studies to strengthen this conclusion are not available.

#### Farmers

In a cross-sectional study, hearing in 60 US farmers from Iowa was compared with hearing in 60 age- and sex-matched nonnoise-exposed controls. The farmers had significantly reduced hearing compared to the controls (Plakke and Dare 1992). Stewart et al. (2003) reported that US part-time farmers had reduced hearing compared to full-time farmers and attributed this to more noisy and low-quality farming machinery in part-time farmers. Renick et al. (2009) examined hearing in 212 American children and young people in agriculture and found that they had a higher hearing threshold and twice as many audiometric notches as a comparable reference population.

A prospective Norwegian study on various occupations and hearing revealed a binaural hearing loss of 5.3 dB in the 3–6-kHz area among farmers, compared with a reference group (teachers) (Engdahl and Tambs 2010). Hwang et al. (2001) also reported an increased risk of hearing loss in farmers associated with noise exposure in an interview survey of 1622 farmers. A report from New Zealand assessed farmers as a high-risk group for occupational hearing loss (Thorne 2006).

In conclusion, farmers seem to be at risk of developing NIHL.

#### Musicians

In 1994, Palin published a review article on hearing in classical musicians (Palin 1994). The author discusses findings from a total of 11 studies published between 1960 and 1992. In several of the studies, the participation rate was low and the measured noise levels are described as “unlikely harmful to hearing”. In spite of this, Palin concludes that the overall opinion is that musicians are at risk of developing NIHL.

Since 1994, several cross-sectional studies of classical musicians have been published (Kahari et al. 2001; Emmerich et al. 2008; Hamdan et al. 2008; Jansen et al. 2009; Pawlaczyk-Luszczynska et al. 2011; Toppila et al. 2011). Few of the studies have measured the exposure levels in the orchestra of the musicians, but some studies show equivalent noise levels of 80–90 dB (A) (Emmerich et al. 2008; Jansen et al. 2009; Pawlaczyk-Luszczynska et al. 2011), and the number of hours of exposure was also carefully recorded (Hamdan et al. 2008). Most of the studies find that the musicians do not get any more hearing loss than the controls. However, there are studies indicating that the hearing loss among musicians is greater than expected for age (Emmerich et al. 2008). Brass players and percussionists may be more affected than other musicians (Pawlaczyk-Luszczynska et al. 2011), and one study suggests a hearing loss in the 6-kHz range for this group (Jansen et al. 2009). Otoacoustic emissions (TEOAE) were recorded in a study of singers. Singers with normal hearing had a lower “signal-to-noise ratio” than controls, which may indicate subclinical cochleae dysfunction (Hamdan et al. 2008). In a follow-up study over 16 years of 56 musicians, male musicians lost 0.7 dB per year in the 3–8-kHz range, while the female musicians lost 0.4 dB per year. The losses were not greater than in a control group (Kahari et al. 2001). Similar results were reported in another study from the Nordic countries, where 135 musicians were examined after 3 and 8 years. After 8 years, the hearing loss was close to normal (Ostri and Parving 1991).

In a cross-sectional study of rock musicians from 1978, the prevalence of hearing impairment was found to be remarkably low (Axelsson and Lindgren 1978). In a follow-up study of 53 Swedish rock musicians 16 years after the initial investigation, the hearing loss was slightly less than expected, despite an equivalent exposure level of 90–105 dB from 20 to 25 h/week (Axelsson et al. 1995). Drummers had a slightly greater hearing loss than other musicians. The author suggests that a positive attitude to the music may have a protective effect on hearing. In another study, a group of drummers was examined by otoacoustic emissions. The amplitudes of DPOAE (6000 Hz) were missing to a greater extent than in the controls, suggesting a hearing loss (Pride and Cunningham 2005).

Other studies of rock/jazz musicians have uncovered equivalent exposure levels from 111 to 129 dB (A) (Kähäri et al. 2003) and 83–90 dB during practice, and 90–96 dB during concerts (McIlvaine et al. 2012). Equivalent levels at 95–108 dB (A) were measured at rock concerts in Sweden (Almstedt et al. 2000). Subjective hearing impairment was reported in 75 % of the musicians (Kähäri et al. 2003). Temporary problems, such as tinnitus and hyperacusis in adolescents attending rock concerts, are also being reported, but an excess risk of permanent hearing loss could not be demonstrated (Almstedt et al. 2000).

The noise exposure level is most pronounced in rock/jazz musicians, who also have more hearing complaints and a greater degree of hearing loss than in the case for classical musicians. A possible elevated risk of hearing loss is quite modest for musicians as a group.

#### Kindergarten employees

Gärding (1980) studied noise and hearing in 17 Swedish nurseries with 79 employees. The equivalent noise exposure was on average 83 dB (A). Employees with more than 11 years of employment heard worse than those with less seniority, but age and noise exposure levels were not adjusted for.

Rubak et al. (2006) compared hearing in a nursery staff with a group not exposed to noise and found that the nursery staff had a normal hearing.

Results from a Norwegian survey revealed normal hearing in a group of 165 nursery workers (Engdahl and Tambs 2010).

Overall, the literature suggests that the noise exposure is too low to cause any hearing loss among nursery staff, and their hearing does not differ from nonexposed controls.

#### Other professions

Lesage et al. conducted a cross-sectional study of hearing in 887 French policemen compared with 805 office workers, using medical records. No noise measurements were available. The risk of hearing loss exceeding 30 dB at 4 kHz was elevated, odds ratio (OR) 1.41 (1.06–1.90), especially for the use of police motorcycles (Lesage et al. 2009).

Hearing in Australian mining workers was tested in 1985–1988 in a cross-sectional study (*N* = 8774). The group had an average daily noise exposure of 90 dB. More than 40 % of them had a hearing loss that gave the right to workers’ compensation. This was less than in the period from 1982 to 1985, where 56 % had a right to compensation. The hearing loss was associated with age and exposure to noise. The use of hearing protection appeared to have some preventive effect (Leigh and Morgan 1990).

Rubak et al. compared hearing in Danish workers in noise hazardous work with nonnoise-exposed and found that the risk of hearing loss >20 dB at 2–4-kHz area was tripled by exposure to noise for more than 20 years. For employees <30 years of age, or who started in noise-exposed work after 1990, there was no increased risk of hearing loss (Rubak et al. 2006).

In a Norwegian prospective study, the mean binaural age-adjusted hearing loss, 3–6 kHz, in men was greatest among woodworkers (11.2 dB), miners (10.9 dB), construction workers (9.2 dB), military personnel (8.2 dB) and farmers (5.3 dB), compared to teachers (Engdahl and Tambs 2010). In younger men and women, the impact of occupational exposure was much smaller.

### Impulse noise and hearing

Exposure to impulse noise among metal workers, workers at a high-voltage transmission station and Swedish military officers have been shown to cause a significant hearing loss (Kamal et al. 1989; Christiansson and Wintzell 1993; McBride and Williams 2000). Most of the hearing loss was in the higher frequencies (3–8 kHz). The hearing loss at 4 kHz was approximately 5–10 dB for those under 30 and 35–40 dB for those between 50 and 60 years of age (Christiansson and Wintzell 1993).

In a Swedish cross-sectional study (Nilsson et al. 1977), hearing was studied in 1492 employees at a shipyard with noise exposure ranging from *L*_Aeq_ = 88 to 94 dB and with a lot of impulse noise, typically 2500 pulses per day with peak noise levels from 105 to 135 dB (A). Despite the fact that almost 90 % used hearing protection, about 60 % were judged to have a noise-induced hearing loss. After individual age correction according to ISO 1999, still about 40 % of them were diagnosed with hearing loss. The authors conclude that the impulse noise in shipyards is particularly damaging to hearing.

Three groups of Finnish metal workers and welders, each with 10 subjects, who had been exposed to impulse noise over a short, medium and long period, respectively, were compared with 12 employees in a cable factory exposed to continuous noise (Mantysalo and Vuori 1984). The longer the duration of impulse noise exposure, the greater the prevalence of hearing loss. It was also concluded that recurrent impulse noise appears to result in permanent hearing loss at frequencies 4 and 6 kHz after a shorter exposure time compared to continuous noise.

In a Norwegian population study (*N* = 51,975), the participants were asked about occupational noise and impulse noise, including shooting (Tambs et al. 2006). It was a clear but moderate effect on hearing among the relatively few women who were exposed to impulse noise. For women over 64 years, a loss of 4–6 dB was found at 3–8 kHz. For men aged 45–64 years, the corresponding hearing loss was 8 dB. Among men older than 64 years, there was a loss of approximately 7 dB in the range 2–8 kHz. Among men younger than 45 years, the hearing loss was 1–3 dB for the frequency range 3–8 kHz. While continuous noise generally resulted in a U-shaped” audiogram with the greatest loss in 3–4 kHz, impulse noise gave a loss in a much larger frequency range, in the oldest group from 2 to 8 kHz.

The referred studies show that impulse noise can cause a significant hearing loss, but do not answer the question whether impulse noise is more damaging to hearing than continuous noise. A main difference is that impulse noise exposure may be extreme and cause an acute hearing loss. Continuous noise will generally generate a slow developing hearing loss.

In a review article, Clifford and Rogers (2009) concludes that impulse noise can cause more damage than the amount of energy calculated would indicate, compared to continuous noise. The reason for this is an overload of both the hair cells and the cellular antioxidant system at high exposure levels. Higher exposure levels may also produce a mechanical damage in the cochlea. Animal studies also indicate that impulse noise is more damaging than continuous noise.

Impulse noise is a particularly important problem in the military, with very high peak exposures, and the degree of protection when using normal hearing protection is therefore limited. The use of hearing protection may also come in conflict with the security during armed missions.

Henderson and Hamernik reported a significantly higher risk of hearing loss for a highly peaked exposure (“high kurtosis”) compared with continuous noise. He also makes note that the USA use a 5-dB correction factor (“trading factor”) compared to the 3 dB in Europe, i.e., the amount of energy is considered to be doubled for every 3-dB increase in European computations compared to 5 dB at US calculations (Henderson and Hamernik 2012). Experts have discussed whether to add a safety margin of 5 dB when estimating the noise exposure of impulse noise at an 8-h basis (Von Gierke et al. 1982; Thorne 2006; Arbejdstilsynet 2007).

### Population-based studies on hearing

Since 1990 when ISO 1999 was published, several major population studies of hearing have been conducted. These have demonstrated a number of factors other than age, gender and noise that affect hearing.

Cruickshanks et al. (1998) conducted a cross-sectional study of 3753 people aged 48–92 years from Beaver Dam, Wisconsin. A hearing loss was defined as a pure tone average (PTA) at 0.5–4 kHz >25 dB in the worst ear. Hearing loss was very common among the elderly, with a prevalence of almost 50 %, and was associated with high age, male gender, low education and income, and occupational noise exposure.

In a later follow-up, the same author examined the cumulative incidence of hearing loss after 2.5, 5 and 10 years (Cruickshanks et al. 2010). The cumulative incidence of hearing loss over a 10-year period was associated with a 5-year increase in age [hazard ratio (HR) 1.81], male gender (HR 2.29), unmarried status (HR 1.29), low education (HR 1.40), noisy occupation (HR 1.34) and a nonsignificant increase in self-reported noise at work (HR 1.16) when adjusted for relevant factors.

Dalton et al. (2001) conducted a new analysis of the “Beaver Dam-material” in 2000 and reported that exposure to leisure noise (woodworking, chainsaw, metalwork) produced a small increase in the risk of hearing loss >25 dB for low-frequency loss (0.5–4 kHz; OR 1.11) and for high-frequency loss (4–8 kHz; OR 1.16) if the exposure was >90 dB (A). The use of musical instruments was associated with a decreased risk of hearing loss. The authors concluded that leisure noise can cause hearing loss in men provided sufficient exposure, but the effect is small.

Data from the “Beaver Dam Offspring Study” (Nash et al. 2011) (*N* = 3285) showed that hearing loss, defined as an average hearing threshold of 25 dB or more for the range of 0.5–4 kHz, was significantly associated with high age, male gender, low education, noisy job, ear surgery and changes in the central vein of the retina of the eye (a measure of vascular changes). The hearing loss associated with a 5-year age increase was 2.4 dB, male gender was associated with a 5.9-dB loss, low versus high education with a 3.6-dB loss, noisy work with a 1.5-dB loss and having undergone surgery to the ear with 8.9-dB loss. A borderline significant association with hearing loss was reported for cardiovascular disease, diabetes, high blood pressure, smoking, lack of exercise and high cholesterol.

Ecob et al. followed individuals born in 1958 being at the age of 45 years in 2003 in an English longitudinal study. Belonging to lower social group (manual vs nonmanual work) was associated with a 1–3-dB hearing loss at 4 kHz in men and <0.7 dB in women, adjusting for relevant factors (Ecob et al. 2008).

Engdahl et al. found in a Norwegian study of 51,975 residents of the county of Nord-Trøndelag (HUNT) that men without any occupational noise exposure had a better hearing of 1–7 dB at 4 kHz in men compared to those exposed, least for the youngest. The corresponding figures were negligible for women (Engdahl et al. 2005). Linking the data of hearing to occupational data from 1970 to 1990 revealed that the occupational hearing loss was greatest, 11 dB, in male woodworkers and miners (Engdahl and Tambs 2010). The hearing loss was greatest in men who were >45 years at the time of the survey. For men <45 years and for women, the occupational hearing loss was much smaller (Tambs et al. 2006).

In an analysis of data from the US Health and Nutrition Survey (NHANES 1999–2002) (*N* = 5742), Agrawal et al. (2009) reported that a hearing loss of 25 dB or more for the range of 0.5–4 kHz was significantly associated with age, gender, Caucasian ethnicity, low education, smoking, noise exposure and risk factors for cardiovascular disease.

Flamme et al. (2011) examined the relationship between hearing and age, gender and ethnicity in 5056 men and women from the same NHANES survey. African-American men had better hearing than white caucasians and men of Mexican ancestry, when relevant external factors were adjusted for. The difference was particularly large for the 3–6-kHz area for ages higher than 30. For a man aged 50–59, the difference at 4 kHz constitutes approximately 10 dB. For women, the differences were only minor.

Based on the NHANES material, Fabry et al. (2011) reported that passive smoking, quantified by measurement of cotinine in urine, was associated with a hearing loss.

Diabetes and age were also reported to predict hearing loss. Diabetics had a greater hearing loss than nondiabetics in the 3–6-kHz range in a major US population survey (NHANES 1999–2004) (Bainbridge et al. 2008).

In 2008, Fransen et al. (2008) conducted a European cross-sectional multicenter study (*N* = 4083) of the impaired hearing due to a number of factors, such as occupational noise, shooting, height, weight, smoking, cholesterol, diabetes, BMI, heart disease, hypertension and pigmentation/eye color. Adjusted for age and gender, hearing loss was associated with occupational noise, high BMI and smoking. Better hearing was associated with moderate alcohol intake and height (a tall person has better hearing than a short one). Factors in other studies that have been associated with hearing loss, such as shooting, high cholesterol, diabetes, exposure to solvents, heart disease, hypertension and pigmentation/eye color, had no significant effect on hearing in this study. Based on the fact that several factors were analyzed simultaneously, the requirements for statistical significance were set high. The authors conclude that age-induced hearing loss may be modestly reduced by the same recommended actions as for the prevention of cardiovascular disease.

Gopinath et al. (2010) examined hearing in 2815 Australian men and women more than 50 years old in a cross-sectional study which was part of the “Blue Mountain Hearing Study”. Adjusting for relevant factors, smoking was associated with an increased risk of hearing loss (OR 1.63), and a moderate alcohol consumption led to a slight protective effect (OR 0.75). At a follow-up 5 years later, they found no effect of alcohol and smoking on new cases of hearing impairment. Food intakes of vitamins A, C and E and beta carotene were measured by an extensive questionnaire. Vitamin intake had no effect on hearing, neither in the first round nor in the 5-year follow-up (Gopinath et al. 2011).

US studies indicate that hearing in the population has improved in recent years. Hoffmann et al. (2010) compared reference data from 1959 to 1962 with data from 1999 to 2004 and found that hearing in the noise-sensitive area (3–6 kHz) has been improved by approximately 5 dB. Less noise exposure, better hearing conservation programs and better management of ear infections were suggested as possible causes.

Similar findings were revealed by Zhan et al. (2010) in a follow-up of hearing data from the Beaver Dam studies from 1993 to 1995 and 2003 to 2005. The prevalence of hearing loss, defined as PTA thresholds at 0.5, 1, 2 and 4 kHz >25 dB, was reduced by 13 % in men and 6 % in women for every 5 years of observation. Lower noise exposure and healthier lifestyle were cited as possible causes.

### Occupational exposure to vibration and hearing

Vibration and vibration-induced white fingers (VWF), with concomitant noise exposure, have for many years been considered as possible risk factors for developing hearing loss.

Iki et al. (1986) examined 74 forest workers, 37 with and 37 without VWF. VWF was associated with greater hearing loss in the noise-sensitive area (3–6 kHz). In a longitudinal study, the same authors reported that individuals with VWF had greater hearing loss in the 2–4-kHz range than controls (Iki et al. 1989).

Similar findings were shown in a cross-sectional study of Romanian miners with (*N* = 84) and without (*N* = 264) VWF (Szanto and Ligia 1999) and in a follow-up of a cohort of 276 Swedish male workshop workers (Pettersson et al. 2012).

Starck et al. (1999) found in a group of Finnish forestry workers (*N* = 199) and shipyard workers (*N* = 171) that smoking, VWF and noise all contributed significantly to the age-adjusted hearing loss. Pyykkö et al. (1986) also reported an association between VWF and hearing in a longitudinal study of 32 forest workers with VWF and 32 matched controls. The difference amounted to about 10 dB at 4 and 8 kHz, but did not increase over time. The same authors reported similar hearing loss (10 dB) associated with VWF in two other studies of forest workers (Pyykkö et al. 1981, 1987), whereas a longitudinal study of 199 forest workers did not reveal any significant effect of vibration on hearing (Pyykkö et al. 1989). In another study of a mixture of miners, metal workers, shipyard workers, forestry workers and patients referred to a clinic, Pyykkö et al. (2007) reported that workers with Raynaud disease were more susceptible to hearing loss at 4 kHz than others.

Virokannas et al. (1994) reported no effect of VWF on hearing in a cross-sectional survey of railway workers (*N* = 117) with significant exposure to noise and vibration.

In an experimental study of healthy young subjects, Zhu et al. (1997) reported that exposure to noise with *L*_Aeq_ >90 dB and vibration gave a greater temporary hearing loss (TTS) than exposure to noise only. Exposure to vibration only had no effect on hearing.

Most of the studies we identified suggest that vibration and VWF are risk factors for getting hearing loss from noise exposure. It is difficult to distinguish vibration from noise since a lot of the noise comes from vibrating tools, such as chainsaws, power tools and pneumatic tools (Burgess and Williams 2006; Thorne 2006). There is still reason to believe that those who have circulation problems, such as VWF, are more likely to develop hearing loss due to noise. Experimental studies support this (Zhu et al. 1997).

### Occupational exposure to chemicals and hearing

During the last 20–25 years, questions have been raised whether chemical substances can cause hearing loss (Johnson and Morata 2010). The focus has especially been on drugs with neurotoxic effects. Most of the research literature is based on experimental work on laboratory animals.

In this review, we have mainly focused on studies in humans, but we have included a few animal experimental studies discussed in review articles (Le Prell et al. 2007; Johnson and Morata 2010; Morata and Johnson 2012).

#### Solvents

In three small cross-sectional studies, Fuente et al. reported that solvent exposure is associated with hearing loss (Fuente and McPherson 2007a, b; Fuente et al. 2009; Fuente and Hickson 2011). The same was reported in studies by Jacobsen et al. (1993) in a Danish cross-sectional study of 3282 men, by Botelho et al. (2009) among 155 Brazilian steel workers and by Kim et al. (2005) among 542 men from the aerospace industry. A Polish cross-sectional study of 3741 men from 24 factories also found that solvent exposure was associated with hearing loss (Dudarewicz et al. 2010). In a cross-sectional study of 393 American apprentices, a 1-year solvent exposure was estimated to cause a hearing loss of 0.6 dB (Seixas et al. 2004).

Chang et al. compared hearing in 58 employees with exposure to noise and toluene with 58 employees with noise exposure only and 58 nonexposed workers. The prevalence of hearing loss >25 dB was higher in the noise + toluene group compared to the noise group and lowest in the nonexposed group (Chang et al. 2006).

Carbon disulfide was shown to reduce hearing in the low frequencies in a study of 346 rayon wool workers, where 105 were exposed to equivalent noise levels of 80–90 dB, 132 were exposed to a combination of noise and carbon disulfide, and the rest were not exposed (Chang et al. 2003). The hearing loss was about 10 dB in both exposed groups compared to the nonexposed.

Sliwinska-Kowalska et al. (2005) reported that the combination of noise and solvents, such as styrene, xylene, n-hexane and toluene, caused a hearing loss in a study of 1117 workers from different industries. Another study of 290 solvent-exposed workers and 213 controls from a plastic boat factory revealed that various combinations of styrene, toluene and noise gave significantly increased hearing loss and that the effects were at least additive, and perhaps synergistic (Sliwinska-Kowalska et al. 2003). In a study of 701 shipyard workers exposed to noise, xylene and toluene, the combination of exposures on hearing was found to be additive (Sliwinska-Kowalska et al. 2004).

In an Australian study from a ceramic factory, solvent-exposed workers got audiometric notches earlier than those who were not solvent exposed, Safia Beshir et al. (2011).

Morata et al. (1993) reported that the combination of noise and solvents increased the hearing loss compared with exposure to noise and solvents separately, i.e., a synergistic effect.

However, no detrimental effect on hearing was found from exposure to styrene in a study of 32 boat builders (Hoffmann et al. 2006) nor in a European population exposed to solvents (Fransen et al. 2008) or in styrene-exposed German ship workers (Triebig et al. 2009).

#### Other chemicals

In a study of 412 Taiwanese steel mill workers, an association between lead in blood and hearing loss was reported, but the study revealed no effect on hearing of exposure to copper, zinc, arsenic and cadmium (Hwang et al. 2009).

Choi et al. (2012) reported an association between cadmium in blood and hearing loss, but not for lead in blood.

Crawford et al. reported a weak correlation between exposure to organophosphates and self-reported hearing loss in a survey of 14,229 pesticide-exposed workers (OR 1.17) (Crawford et al. 2008).

#### Possible mechanisms

There are numerous studies on humans showing that various chemicals may affect hearing. The exposure data on noise and chemicals are of low quality in most studies, and it is therefore difficult to conclude on a dose–response relationship.

In a review article, Morata and Johnson (2012) have described a number of animal studies showing dose–response relationship. Several solvents, such as styrene, toluene, xylene, ethylbenzene, trichloroethylene, n-hexane, jet fuel, white spirit and other solvent mixtures, have ototoxic properties in humans and affect both cochlear and more central nervous structures (Johnson and Morata 2010). Ototoxic properties are also evidenced by exposure to lead, carbon monoxide and pesticides, such as organophosphates (Burgess and Williams 2006). There is experimental evidence from animal studies that the effects of noise and solvents are synergistic with regard to hearing loss (Morata and Johnson 2012), and this may also be the case in human studies (Jacobsen et al. 1993; Morata et al. 1993; Kim et al. 2005; Fuente and McPherson 2007a, b; Botelho et al. 2009; Fuente et al. 2009; Fuente and Hickson 2011). The damage to the cochlea of ototoxic substances is similar to that from exposure to noise and may explain the synergistic effect.

In addition, it appears that age may modify the ototoxic effect. Young laboratory animals appear to be more vulnerable than older ones with respect to hearing loss from both noise and chemicals. Whether this also applies to exposure to chemicals or noise in humans is more uncertain (Johnson and Morata 2010).

In summary, the chemicals best documented to have an effect on hearing in humans at exposures around the occupational exposure limit (OEL) are styrene, toluene, lead, mercury, carbon disulfide and carbon monoxide (Johnson and Morata 2010).

Several medications have ototoxic properties. Most notable is the cancer drug, cisplatin, which has a fairly strong ototoxic effect. Another group of ototoxic drug is aminoglycosides. Aspirin may also cause hearing loss (Johnson and Morata 2010).

The US National Institute of Occupational Safety and Health (NIOSH) and the American College of Occupational and Environmental Medicine (ACOEM) have recommended that exposure to ototoxic drugs and chemicals must be taken into account when risk assessments and hearing conservation programs are being made (Johnson and Morata 2010; Kirchner et al. 2012). The US Occupational Safety and Health Administration (OSHA) recommends regular hearing testing of workers exposed to ototoxic substances. In Australia and New Zealand, compensation for occupational hearing loss caused by ototoxic drugs and chemicals may be granted (Johnson and Morata 2010).

The relationship between ototoxic drugs and chemicals and hearing loss is far from fully examined, but such exposures must be included in the assessment of possible occupational hearing loss in workers and in the design of preventive measures (Johnson and Morata 2010; Kirchner et al. 2012).

### Leisure-time noise and hearing

There is a common perception that leisure noise from music players, concert participation, hunting and shooting, and noisy tools, such as chain saws and drills, may damage hearing.

Tambs et al. (2003) reported an effect on hearing of the use of firearms, with a resulting hearing loss of 7–8 dB, but not of music (attendance at concerts or disco visit, playing in a band or listening to portable music players) in a Norwegian population study. In a study of otoacoustic emission (OAE) from the same study, Engdahl did not find any effect of music on hearing (Engdahl and Tambs 2002).

In a population-based study from the USA (Beaver Dam) (*N* = 3571), Dalton et al. (2001) reported that leisure noise (woodworking, metalworking, chainsaw, music, etc.) gave a slightly increased risk of a hearing loss (OR 1.1) if the equivalent noise exposure level was >90 dB (A). The use of a musical instrument was associated with a decreased risk of hearing loss. In a cross-sectional study of 3510 nuclear power-plant workers, Dement et al. (2005) did not find any effect of leisure-time noise on hearing.

In a summary report, Thorne (2006) argues that music to the ear can cause damage to hearing if the dose is large enough, but for most purposes, the volume and the length of exposure are not high enough to have any detrimental effect on hearing. Noise from firearms, however, can be strong enough to damage hearing. Dobie (2008) also highlights the effect of shooting noise on hearing. Clark (1991) is of the same opinion in a review article about leisure-time noise and hearing and concludes that firearms are the main problem as a noise source, not music. Zhao et al. mention a possible problem with new types of music players with great battery life found in mobile phones, etc., that makes long-term exposure to music possible. Longitudinal studies are lacking (Zhao et al. 2010). Harrison (2012) expressed concern about the number of children who have a hearing loss, possibly due to noise from music players and other noise sources. Others have also expressed concern that leisure-time noise may damage hearing, but admit that the evidence for this is scarce (Scenihr 2008; Basner et al. 2013).

In summary, it appears that the possible harmful effect of firearms on hearing is well documented. Other types of leisure-time noise are less likely to result in a hearing loss sufficiently great to produce detectable evidence on a group level. On an individual level, however, hearing loss may occur given a sufficient exposure time and level.

### Other factors that may affect hearing

Several studies have indicated a relationship between hearing loss and smoking (Barone et al. 1987; Mizoue et al. 2003; Uchida et al. 2005; Wild et al. 2005; Dudarewicz et al. 2010). An association between hearing loss and high levels of blood lipids has also been reported. Axelsson and Lindgren (1985) examined hearing in 78 persons with cholesterol >7.0 mmol/L and 75 with cholesterol <5.6 mmol/L and reported that high cholesterol was associated with a hearing loss. Fuortes et al. (1995) reported that hearing loss among 665 workers at an American university was associated with high cholesterol and high blood pressure. Chang et al. (2007) reported a weak association between high blood triglycerides and hearing loss (OR 1.28), but not for high cholesterol (OR 0.95) among 4071 men and women who underwent health checkups.

The incidence of type 2 diabetes was also reported to be associated with a hearing loss (Ishii et al. 1992).

The hypothesis that factors influencing the microcirculation can lead to hearing loss is plausible since the organ of Corti has a significant blood flow and a hearing loss is associated with decreased local circulation along with the formation of free radicals. Findings from several surveys are somewhat contradictory. Adjustments for possibly relevant factors seem to reduce the impact of cholesterol, triglycerides, high blood pressure, diabetes and heart disease on hearing. However, it appears that smoking remains as a factor of some importance for the impairment of hearing. This can possibly be explained by a general inflammatory process in the body resulting from smoking, which also may affect the inner ear. It is therefore unclear whether prevention efforts aimed at cardiovascular disease will have any effect on hearing (Fransen et al. 2008).

### Summary of results

Table 1 shows a summary of the results of the literature review and the level of evidence. Increasing age is strongly related to hearing loss. Men lose more hearing with age than women. Hereditary factors explain why the variation between individuals is great. Ear disorders may affect hearing, while cardiovascular risk factors have only a minor impact on hearing. Workers in industry, shipbuilding, construction industry, military and farmers have the highest risk of hearing loss. The risk is primarily related to the degree of noise exposure and the use of hearing protection. Continuous noise and impulse noise can damage hearing if the exposure is high enough. Impulse noise is probably more harmful than continuous noise at the same level of noise exposure (*L*_ex_, _8h_). It is well documented that shooting may affect hearing, while the effect of other types of leisure noise is uncertain. Use of hearing protection and noise reduction measures protects against NIHL. Certain drugs may cause a significant hearing loss. The impact on hearing of other chemical substances and vibration is probably of limited importance.

**Table 1:** Risk factors for hearing loss

	Risk	Evidence	Comments
Personal factors			
Age	+++	***	High age is strongly related to hearing loss
Male sex	++	***	Men lose more hearing than women
Hereditary conditions	++	***	Explains a great part of the individual variation in hearing loss
Socioeconomic factors	+	**	Low social class, income and education related to reduced hearing
Ethnicity	+	**	White Caucasians lose more hearing than Afro-Americans
Health factors			
Ear disease	++	***	
Cardiovascular disease	+	*	
Hypertension	+	*	
Diabetes	+	*	
Smoking	+	*	
Cholesterol	?	*	
Triglycerides	?	*	
Occupations			
Industrial workers	++	**	Depending on noise exposure level and use of protection
Shipyard workers	++	**	
Construction workers	++	**	
Offshore workers	+	*	
Professional divers	+	**	
Fire fighters	+	**	
Military workers	++	**	
Civil aviation workers	+	**	
Railway workers	+	**	
Farmers	++	**	
Musicians	+	**	
Kindergarten employees	+	*	Probably too low noise exposure
Noise exposure			
Continuous noise	+/+++	***	High risk with unprotected noise exposure *L*_ex, 8h_ > 90 dB. Low risk <85 dB
Impulse noise	+++	***	
Gunfire	++	***	
Leisure-time noise	+	**	Probably of minor importance at a group level
Hearing protection	−	**	
Other exposures			
Vibration	+	*	Vibration may increase the NIHL
Chemicals	+	*	Styrene, CS_2_, toluene, lead, mercury and CO
Medication	+/+++	***	Cisplatin, aminoglycosides

Risk assessment: +++, severe risk; ++, moderate risk; +, low risk; ?, uncertain risk; −, reduced risk

Level of evidence: ***, high; **, medium; *, low

## Discussion

This is a systematic review of a large number of studies within this field of research and included systematic assessment of the quality of eligible studies. Many of the included studies are cross-sectional, which may be considered a weakness. We regarded it necessary to include these studies to get a good enough description of occupations and hearing loss and because the majority of the population studies are cross-sectional. The studies are usually based on measurements of noise and hearing, not self-report. Thus, both exposure and outcome data come from objective data sources. Since hearing impairment rarely leads to relocation of the worker to another job or loss of employment, this probably represents a minor problem. We therefore believe that cross-sectional studies on noise and hearing provide relatively valid data.

The diversity of the outcome measures, particularly the use of different definitions of NIHL, made comparisons between studies more difficult. We cannot exclude the possibility of publication bias. Studies with positive results are often easier to publish than studies with nonpositive results. We found articles from other sources that had not been identified in the literature search, which might imply that we may have missed some literature, in spite of our choice of a sensitive rather than a specific search strategy.

Population studies generally have high-quality data on outcomes, such as hearing, and also fairly good quality on possible confounding or modifying factors, such as smoking, heart disease and blood pressure, but the noise exposure data are usually of a lower quality. Population studies show that age is by far the most important predictor of hearing loss. By age 60, age-related hearing loss (in the 3–6-kHz range) is approximately 30–40 dB for males and 20 dB for females. Determining whether hearing loss exceeds the age-expected decline is made feasible by comparison with age-related hearing levels for populations who have not been exposed to noise. At the group level, hearing loss in noise-exposed workers that exceeds the age norm can be ascribed to occupational noise exposure and other exposures and factors that may cause a hearing loss. On an individual level, however, it is not possible to distinguish between a hearing loss due to aging, genetic predisposition and noise exposure. Impulse noise is probably more damaging to hearing than continuous noise and can, if the exposure level is high enough, lead to a permanent hearing loss.

Other factors, in addition to noise, may have an impact on hearing. Population studies show that men are more likely to experience hearing loss than women, and genetic factors also play an important role. Moreover, social economic background and ethnicity are of importance (African-Americans experience less hearing loss than Caucasians). Smoking, coronary heart disease, diabetes, high blood pressure and other risk factors for heart disease, such as elevated level of cholesterol, seem to lead to hearing damage, but here the research findings are more uncertain, and there are reasons to expect some publication bias. Exposure to chemicals (e.g., solvents, lead) and certain medications may cause hearing loss, and it appears that concurrent vibration may enhance the harmful effects of noise on hearing. Noisy leisure activities, especially use of firearms, can also lead to hearing loss. Leisure noise from other sources (e.g., iPods, concerts, home-repair tools) appears to have a small effect on hearing loss in population studies, but may still be harmful at the individual level if the exposure is high enough.

Studies on hearing in various professions suggest that hearing loss due to workplace noise was a significant problem in the 1960s and 1970s in industrialized countries, whereas hearing loss has been a less frequent problem in subsequent decades. The reduced occurrence of hearing loss is probably a result of decreased noise exposure, improved regulation and use of protective equipment, but the evidence for this is still limited (Verbeek et al. 2012). This positive trend does not apply to developing countries, where exposure to high levels of noise at work is still significant. As of today, groups of higher risk are found in the armed forces, the engineering industry, building and construction, and agriculture. Employees who seem to have little or no risk of harmful noise exposure at work are people employed in school, day care, transportation, musicians, police and firefighters.

## Electronic supplementary material

Supplementary material 1 (DOCX 24 kb)
