# Supplementary material for: Occupational noise exposure and hearing: a systematic review
Source: Int Arch Occup Environ Health. 2015 Aug 7;89:351–72. doi: 10.1007/s00420-015-1083-5 (PMC4786595; doi:10.1007/s00420-015-1083-5)
Supplement: Supplementary file 1 — Supplementary material 1 (DOCX 24 kb) [file 420_2015_1083_MOESM1_ESM.docx]

## Supplement 1: Search strategies

## OVID Medline

|  |  |
| --- | --- |
| 1. | exp Noise/ |
| 2. | exp Acoustics/ |
| 3. | noise?.mp. |
| 4. | acoustic?.tw. |
| 5. | or/1-4 |
| 6. | exp Occupational Exposure/ |
| 7. | exp Occupational Diseases/ |
| 8. | Occupational Health/ |
| 9. | Occupational Medicine/ |
| 10. | Accidents, Occupational/ or Occupational Injuries/ |
| 11. | Occupations/ |
| 12. | exp Occupational Groups/ |
| 13. | exp Work/ |
| 14. | Women, Working/ |
| 15. | exp Employment/ |
| 16. | Health Occupations/ |
| 17. | exp Industry/ |
| 18. | exp Environmental Exposure/ |
| 19. | occupation$.mp. |
| 20. | industry.mp. |
| 21. | employment.mp. |
| 22. | (working not (working group or working memory)).ti. |
| 23. | worker?.tw. |
| 24. | personnel.mp. |
| 25. | staff.mp. |
| 26. | (industrial adj3 (hygiene or health or medicine or exposure?)).tw. |
| 27. | employee?.mp. |
| 28. | workplace?.tw. |
| 29. | worksite?.tw. |
| 30. | workforce.tw. |
| 31. | at work.tw. |
| 32. | work.ti,hw. |
| 33. | ((work$ or job) adj3 (site? or place? or location? or environment$ or related or condition? or health)).tw. |
| 34. | workrelated.tw. |
| 35. | vocation$.ti,hw. |
| 36. | or/6-35 |
| 37. | 5 and 36 |
| 38. | exp Health/ |
| 39. | exp Disease/ |
| 40. | exp Morbidity/ |
| 41. | health.mp. |
| 42. | exp Occupational Diseases/ |
| 43. | disease?.mp. |
| 44. | morbidity.tw. |
| 45. | illness$.tw. |
| 46. | disorder?.mp. |
| 47. | or/38-46 |
| 48. | 5 and 47 |
| 49. | exp noise/ae |
| 50. | 48 or 49 |
| 51. | 37 and 50 |
| 52. | Hearing/ |
| 53. | exp Hearing Disorders/ |
| 54. | exp Hearing Tests/ |
| 55. | hearing.tw. |
| 56. | exp hearing loss/ |
| 57. | audition.tw. |
| 58. | auditory perception.tw. |
| 59. | audiogram?.tw. |
| 60. | tinnitus.tw. |
| 61. | deafness.tw. |
| 62. | deaf.tw. |
| 63. | or/52-62 |
| 64. | 37 and 63 |
| 65. | 51 or 64 |
| 66. | exp Heart Diseases/ |
| 67. | Cardiovascular Diseases/ |
| 68. | Blood Pressure/ |
| 69. | exp hypertension/ or exp hypotension/ |
| 70. | exp Fatigue/ |
| 71. | Stress, Psychological/ or Stress, Physiological/ |
| 72. | Burnout, Professional/ |
| 73. | ((heart or cardiac or cardiovascular or vascular or myocardial) adj2 (disease? or disorder? or symptom?)).tw. |
| 74. | blood pressure.tw. |
| 75. | fatigue.tw. |
| 76. | tiredness.tw. |
| 77. | stress.tw. |
| 78. | burnout.tw. |
| 79. | or/66-78 |
| 80. | exp Fetal Development/ |
| 81. | exp Congenital Abnormalities/ |
| 82. | exp Pregnancy Complications/ |
| 83. | exp Prenatal Injuries/ |
| 84. | birth defect?.tw. |
| 85. | ((fetal or fetus or foetal or foetus or prenatal or congenital) adj2 (injur$ or exposur$ or diagnos$ or abnormal$ or complication? or development$ or defect? or maturity or disorder? or growth)).tw. |
| 86. | (pregnancy adj2 (disorder? or complication?)).tw. |
| 87. | or/80-86 |
| 88. | 79 or 87 |
| 89. | 37 and 88 |

## OVID Embase

1. exp noise/

2. exp noise pollution/

3. noise injury/

4. acoustics/

5. noise?.mp.

6. acoustic?.tw.

7. or/1-6

8. exp "occupation and occupation related phenomena"/

9. exp occupational disease/

10. exp named groups by occupation/

11. exp work/

12. construction work/

13. worker.hw. not exp "worker(insect)"/

14. working.hw. not (exp working animal/ or exp working memory/)

15. worker/

16. exp occupation/

17. exp occupational medicine/

18. exp "industry and industrial phenomena"/

19. "occupational health and industrial medicine".ec.

20. industry.mp.

21. occupation$.mp.

22. employment.mp.

23. (working not (working group or working memory)).ti.

24. worker?.tw.

25. personnel.mp.

26. staff.mp.

27. (industrial adj3 (hygiene or health or medicine or exposure?)).tw.

28. employee?.mp.

29. workplace?.tw.

30. worksite?.tw.

31. workforce.tw.

32. at work.tw.

33. work.ti,hw.

34. ((work$ or job) adj2 (site? or place? or location? or environment$ or related or condition? or health)).tw.

35. workrelated.tw.

36. vocation$.ti,hw.

37. or/8-36

38. 7 and 37

39. exp health/ or exp health status/

40. diseases/ or exp "general aspects of disease"/ or exp physical disease/

41. environmental health/

42. exp health statistics/

43. exp health survey/

44. exp morbidity/

45. morbidity.tw.

46. health.mp.

47. disease?.mp.

48. illness$.tw.

49. disorder?.mp.

50. or/39-49

51. 7 and 50

52. 37 and 51

53. exp hearing/

54. exp hearing disorder/

55. exp hearing test/

56. hearing.tw.

57. audition.tw.

58. audiometr$.tw.

59. auditory perception.tw.

60. audiogram?.tw.

61. tinnitus.tw.

62. deafness.tw.

63. deaf.tw.

64. or/53-63

65. 38 and 64

66. 52 or 65

67. exp cardiovascular disease/

68. exp blood pressure/

69. exp fatigue/

70. exp stress/

71. acoustic stress/

72. ((heart or cardiac or cardiovascular or vascular or myocardial) adj2 (disease? or disorder? or symptom?)).tw.

73. blood pressure.tw.

74. fatigue.tw.

75. tiredness.tw.

76. stress.mp.

77. burnout.tw.

78. hypertension.tw.

79. hypotension.tw.

80. or/67-79

81. exp fetus development/

82. exp congenital disorder/

83. exp pregnancy disorder/

84. exp prenatal disorder/

85. exp prenatal development/

86. prenatal growth/

87. prenatal mortality/

88. prenatal exposure/

89. birth defect?.tw.

90. ((fetal or fetus or foetal or foetus or prenatal or congenital) adj2 (injur$ or exposure$ or diagnos$ or abnormal$ or complication? or development$ or defect? or maturity or disorder? or growth)).tw.

91. (pregnancy adj2 (disorder? or complication?)).tw.

92. or/81-91

93. 80 or 92

94. 38 and 93

95. 66 or 94

96. limit 95 to embase

## Web of Science

| # 55 | #54 OR #39 OR #28 |
| --- | --- |
| # 54 | #53 AND #21 |
| # 53 | #52 OR #48 |
| # 52 | #51 OR #50 OR #49 |
| # 51 | Topic=(pregnancy near/2 (disorder$ or comlication$)) |
| # 50 | Topic=((fetal or fetus or foetal or foetus or prenatal or congenital) near/2 (injur* or exposur* or diagnos* or abnormal* or complication$ or development* or defect$ or maturity or disorder? or growth)) |
| # 49 | Topic=(birth near/2 defect$) |
| # 48 | #47 OR #46 OR #45 OR #44 OR #43 OR #42 OR #41 OR #40 |
| # 47 | Topic=(hypotension) |
| # 46 | Topic=(hypertension) |
| # 45 | Topic=(burnout) |
| # 44 | Topic=(stress) |
| # 43 | Topic=(tiredness) |
| # 42 | Topic=(fatigue) |
| # 41 | Topic=("blood pressure") |
| # 40 | Topic=((heart or cardiac or cardiovascular or vascular or myocardial) near/2 (disease$ or disorder$ or symtom$)) |
| # 39 | #38 AND #21 |
| # 38 | #37 OR #36 OR #35 OR #34 OR #33 OR #32 OR #31 OR #30 OR #29 |
| # 37 | Topic=(deaf) |
| # 36 | Topic=(deafness) |
| # 35 | Topic=("acoustic stimulation") |
| # 34 | Topic=(tinnitus) |
| # 33 | Topic=(audiogram$) |
| # 32 | Topic=(auditory near/2 perception) |
| # 31 | Topic=(audition) |
| # 30 | Topic=(audiometr*) |
| # 29 | Topic=(hearing) |
| # 28 | #27 AND #21 |
| # 27 | #26 OR #25 OR #24 OR #23 OR #22 |
| # 26 | Topic=(morbidity) |
| # 25 | Topic=(disorder$) |
| # 24 | Topic=(illness*) |
| # 23 | Topic=(disease$) |
| # 22 | Topic=(health) |
| # 21 | #20 AND #3 |
| # 20 | #19 OR #18 OR #17 OR #16 OR #15 OR #14 OR #13 OR #12 OR #11 OR #10 OR #9 OR #8 OR #7 OR #6 OR #5 OR #4 |
| # 19 | Topic=(vocation*) |
| # 18 | Topic=(workrelated) |
| # 17 | Topic=((work* or job) near/2 (site$ or place$ or location$ or environment* or related or condition$ or health)) |
| # 16 | Topic=(job$) |
| # 15 | Topic=("at work") |
| # 14 | Topic=(workforce) |
| # 13 | Topic=(worksite$) |
| # 12 | Topic=(workplace$) |
| # 11 | Topic=(employee$)  Databases=SCI-EXPANDED, SSCI Timespan=All years |
| # 10 | Topic=(staff) |
| # 9 | Topic=(personnel) |
| # 8 | Topic=(worker$) |
| # 7 | TI=(working not ("working group$" or "working memory")) |
|  |  |
| # 6 | Topic=(employment) |
| # 5 | Topic=(industr*) |
| # 4 | Topic=(occupation*) |
| # 3 | #2 OR #1 |
| # 2 | Topic=(acoustic*) |
| # 1 | Topic=(noise$) |

## Scopus

(((((TITLE-ABS-KEY(noise)) OR (TITLE-ABS-KEY(acoustic*))) AND ((TITLE-ABS-KEY(occupation* OR employment OR industry)) OR (TITLE(working AND NOT ("working group" OR "working memory"))) OR (TITLE-ABS-KEY(worker OR personnel OR staff OR employee OR workplace OR worksite OR workforce)) OR (TITLE-ABS-KEY(industr* W/3 (hygiene OR health OR medicine OR exposur*))) OR (TITLE-ABS-KEY("at work" OR job OR workrelated OR vocation*)) OR (TITLE-ABS-KEY((work* OR job) W/2 (site OR place OR location OR environment* OR related OR condition OR health))))) AND (TITLE-ABS-KEY(health OR disease OR illness OR disorder OR morbidity))) OR ((((TITLE-ABS-KEY(noise)) OR (TITLE-ABS-KEY(acoustic*))) AND ((TITLE-ABS-KEY(occupation* OR employment OR industry)) OR (TITLE(working AND NOT ("working group" OR "working memory"))) OR (TITLE-ABS-KEY(worker OR personnel OR staff OR employee OR workplace OR worksite OR workforce)) OR (TITLE-ABS-KEY(industr* W/3 (hygiene OR health OR medicine OR exposur*))) OR (TITLE-ABS-KEY("at work" OR job OR workrelated OR vocation*)) OR (TITLE-ABS-KEY((work* OR job) W/2 (site OR place OR location OR environment* OR related OR condition OR health))))) AND ((TITLE-ABS-KEY(hearing OR audiometr* OR audition OR "auditory perception" OR audiogram)) OR (TITLE-ABS-KEY(tinnitus OR "acoustic stimulation" OR deafness OR deaf))))) OR ((((TITLE-ABS-KEY(noise)) OR (TITLE-ABS-KEY(acoustic*))) AND ((TITLE-ABS-KEY(occupation* OR employment OR industry)) OR (TITLE(working AND NOT ("working group" OR "working memory"))) OR (TITLE-ABS-KEY(worker OR personnel OR staff OR employee OR workplace OR worksite OR workforce)) OR (TITLE-ABS-KEY(industr* W/3 (hygiene OR health OR medicine OR exposur*))) OR (TITLE-ABS-KEY("at work" OR job OR workrelated OR vocation*)) OR (TITLE-ABS-KEY((work* OR job) W/2 (site OR place OR location OR environment* OR related OR condition OR health))))) AND (((TITLE-ABS-KEY((heart OR cardiac OR cardiovascular OR vascular OR myocardial) W/2 (disease OR disorder OR symptom))) OR (TITLE-ABS-KEY("blood pressure" OR fatigue OR tiredness OR stress OR burnout OR hypertension OR hypotension))) OR ((TITLE-ABS-KEY((fetal OR fetus OR foetal OR foetus OR prenatal OR congenital) W/2 (injur* OR exposur* OR diagnos* OR abnormal* OR complication OR development* OR defect OR maturity OR disorder OR growth))) OR (TITLE-ABS-KEY("birth defect")) OR (TITLE-ABS-KEY(pregnancy W/2 (disorder OR complication))))))

## ProQuest Health and Safety Science Abstracts

Searched for:

(((noise*1 OR acoustic*) AND ((industry OR occupation* OR employment OR worker*1) OR ti(working NOT ("working group" OR "working memory")) OR (personnel OR staff OR employee*1 OR workplace*1 OR worksite*1 OR workforce) OR (industrial NEAR/3 (hygiene OR health OR medicine OR exposure*)) OR "at work" OR ti(work) OR ((work* OR job) NEAR/2 (site*1 OR place*1 OR location*1 OR environment* OR related OR condition*1 OR health)) OR workrelated OR workrelated OR vocation*)) AND

(health OR disease*1 OR illness* OR disorder*1 OR morbidity)) OR ((((noise*1 OR acoustic*) AND ((industry OR occupation* OR employment OR worker*1) OR ti(working NOT ("working group" OR "working memory")) OR (personnel OR staff OR employee*1 OR workplace*1 OR worksite*1 OR workforce) OR (industrial NEAR/3 (hygiene OR health OR medicine OR exposure*)) OR "at work" OR ti(work) OR ((work* OR job) NEAR/2 (site*1 OR place*1 OR location*1 OR environment* OR related OR condition*1 OR health)) OR workrelated OR workrelated OR vocation*)) AND (health OR disease*1 OR illness* OR disorder*1 OR morbidity)) OR (hearing OR audiometr* OR audition OR audiogram*1 OR tinnitus) OR "auditory perception" OR "acoustic stimulation" OR (deafness OR deaf)) OR ((((heart OR cardiac OR cardiovascular OR vascular OR myocardial) NEAR/2 (disease*1 OR disorder*1 OR symptom*1)) OR "blood pressure" OR (fatigue OR tiredness OR stress OR burnout OR hypertension OR hypotension)) AND ((noise*1 OR acoustic*) AND ((industry OR occupation* OR employment OR worker*1) OR ti(working NOT ("working group" OR "working memory")) OR (personnel OR staff OR employee*1 OR workplace*1 OR worksite*1 OR workforce) OR (industrial NEAR/3 (hygiene OR health OR medicine OR exposure*)) OR "at work" OR ti(work) OR ((work* OR job) NEAR/2 (site*1 OR place*1 OR location*1 OR environment* OR related OR condition*1 OR health)) OR workrelated OR workrelated OR vocation*))) OR (((noise*1 OR acoustic*) AND ((industry OR occupation* OR employment OR worker*1) OR ti(working NOT ("working group" OR "working memory")) OR (personnel OR staff OR employee*1 OR workplace*1 OR worksite*1 OR workforce) OR (industrial NEAR/3 (hygiene OR health OR medicine OR exposure*)) OR "at work" OR ti(work) OR ((work* OR job) NEAR/2 (site*1 OR place*1 OR location*1 OR environment* OR related OR condition*1 OR health)) OR workrelated OR workrelated OR vocation*)) AND ("birth defect*1" OR ((fetal OR fetus OR foetal OR foetus OR prenatal OR congenital) NEAR/2 (injur* OR exposur* OR diagnos* OR abnormal* OR complication*1 OR development* OR defect*1 OR maturity OR disorder*1 OR growth)) OR (pregnancy NEAR/2 (disorder*1 OR complication*1))))
